# Factors Associated with Shooting Accuracy and Wounding Rate of Four Managed Wild Deer Species in the UK, Based on Anonymous Field Records from Deer Stalkers

**DOI:** 10.1371/journal.pone.0109698

**Published:** 2014-10-15

**Authors:** Nicholas J. Aebischer, Christopher J. Wheatley, Hugh R. Rose

**Affiliations:** 1 Game & Wildlife Conservation Trust, Fordingbridge, Hampshire, United Kingdom; 2 British Deer Society, Fordingbridge, Hampshire, United Kingdom; Institut Pluridisciplinaire Hubert Curien, France

## Abstract

The amount of wounding during routine culling is an important factor in the welfare of wild deer. Little information exists on factors determining shooting accuracy and wounding rates under field conditions in the UK. In this study, 102 anonymous stalkers collected data on the outcomes and circumstances of 2281 shots. Using hot-deck imputation and generalised linear mixed modelling, we related the probability that a shot hit its target, and the probability that the shot killed the deer if it was hit, to 28 variables describing the circumstances of the shot. Overall, 96% of deer were hit, of which 93% were killed outright. A reduced probability of hitting the target was associated with an uncomfortable firing position, too little time available, shooting off elbows or freehand, taking the head or upper neck as point of aim, a heavily obscured target, a distant target, shooting at females, lack of shooting practice and a basic (or no) stalker qualification. An increase in the likelihood of wounding was associated with an uncomfortable firing position, shooting with insufficient time, a distant target (only when time was not sufficient), a bullet weight below 75 grains, a target concealed in thicket or on the move and an area rarely stalked. To maximise stalking success and deer welfare, we recommend that stalkers ensure a comfortable firing position, use a gun rest, aim at the chest, use bullets heavier than 75 grains, avoid taking a rushed shot, shoot a distant animal only if there is plenty of time, fire only when the target is stationary, avoid shooting at an obscured animal, take care when the ground is unfamiliar, and do shooting practice at least once a month. The high miss rate of basic-level stalkers suggests that training should include additional firing practice under realistic shooting conditions.

## Introduction

The wild deer currently found in the UK are a mix of native and naturalised species [1], [2]. The two native species are the red deer *Cervus elaphus* and roe deer *Capreolus capreolus*. The fallow deer *Dama dama* went extinct in Britain during the last Ice Age, and was re-established by the Normans for hunting in the 11th century. The three remaining species, sika deer *Cervus nippon*, muntjac *Muntiacus reevesii* and Chinese water deer *Hydropotes inermis* were introduced from the Far East in the late 19th and early 20th centuries.

Nationwide monitoring of deer abundance by the British Trust for Ornithology [3] and of numbers of shot animals by the Game & Wildlife Conservation Trust [4] has shown that all species except possibly Chinese water deer are increasing in numbers and range. Such increases are not without problems (e.g. [5]). Deer can cause damage to agricultural and horticultural crops, woodland and forestry, and gardens and parks [6], [7], [8], [9], [10], [11]. They can pose a health hazard by acting as reservoirs for human and livestock diseases [12], [13], [14], [15]. They can also pose a safety hazard, for example by colliding with vehicles on roads [16], [17], [18].

Because major mammalian predators were eliminated from the UK long ago [2], human management of wild deer populations is essential. Fencing and deterrents can be used in some circumstances to limit deer access to vulnerable crops or roads, but culling using a high-powered rifle is generally accepted as the most effective and humane method of controlling wild deer numbers [19]. A guiding principle in UK deer management is that increasing intervention imposes increasing responsibility for the welfare of the deer [20]. In the case of managing deer by shooting, good welfare equates to killing the quarry as soon as possible (ideally with a single shot) and taking every effort to avoid prolonged suffering of an inadvertently injured animal. To this end, UK legislation lays down minimum standards for the firearms and bullets that can be used to shoot deer, prescribing for example the use of soft- or hollow-nosed (expanding) bullets because they are more likely to kill quickly than hard-nosed bullets [21]. In parallel, the Deer Commission Scotland and the Deer Initiative Partnership (England and Wales) have issued best-practice guidelines for deer management.

In pursuit of high standards in the humane management of wild deer in the UK, a knowledge-based qualification has been devised enabling deer shooters (known as ‘stalkers’) to demonstrate their knowledge and competence. Deer Stalking Certificate 1 is a basic mainly knowledge-based qualification that includes practical assessment of shooting skill and field safety. Deer Stalking Certificate 2 requires documented evidence to be submitted by an experienced witness of the candidate's practical competence in the field, demonstrated over three complete deer-stalking occasions. Some older stalkers may also hold a British Deer Society Advanced Stalker Certificate (an award now discontinued), which was gained by attending a five-day course and passing an assessment principally concerned with deer management, but also including a more difficult shooting test and other relevant training, e.g. wounded deer retrieval techniques.

A review of current and future deer management options [22] reported that the annual deer cull in England was from about 70,000 to over 100,000 animals, virtually all culled by rifle. It reported tentatively that about 5% of deer culled may require a second shot (M. Squire pers. comm. from British Deer Society data), and that about 2% of deer shot may escape wounded [23]. Another study into a single species, red deer, discovered that 14% of culled animals had received more than one shot [24]. These studies of the effectiveness of shooting by rifle were few and limited in scope. They shed little light on what could be done to minimise wounding and improve welfare.

In 2005, the British Deer Society (BDS) initiated a research project to identify the important factors contributing to accuracy when shooting deer. The aim was to establish a scientific basis for refining the instruction on BDS Deer Stalking Certificate training courses and improve BDS advice to deer stalkers, so as to improve deer welfare during culling.

This study evaluated the accuracy of shooting in two ways, first through the probability that a target deer was hit by the shot, and second through the probability that the shot killed the deer if it was hit. It seeks to determine how changes in these probabilities were associated with characteristics of the stalker, the firearm, the shooting position of the stalker, the posture of the animal, the habitat and the weather.

## Materials and Methods

### Data collection

Data collection depended on stalkers in the UK providing detailed information about the circumstances of each shot fired at a wild deer. A pilot survey was carried out to test the usability and effectiveness of the draft data collection forms. The set of variables on which information was requested, the layout of the forms and the accompanying instructions were finalised based on feedback from the participants. The main survey was based on two data capture forms with instruction sheets describing the variables and categories for which information was requested. The first (Form S1) recorded details of each shot fired at a deer, including the conditions under which the shot was taken. The second (Form S2) recorded details of each deer fired at, the environmental conditions and the outcome of the shot.

By advertising to the membership of the British Deer Society and personal contacts, a register of stalkers willing to provide data over the course of a year was established and information covering the age, experience, qualifications and employment profile of each was recorded. In order to gain an initial assessment of their previous activity, their stalking data for the previous five years (1999–2003) was requested. The volunteers were asked to fill in the forms mentioned above each time that they participated in deer culling. The data entered on the forms depended on the subjective judgement and honesty of the stalker. It was thought that the best way to obtain honest data was by asking for anonymous returns, so volunteers were issued with an anonymous registration number that was then used for all future data consolidation. If a stalker had to be contacted over a query, this was done via the project organiser who was the sole person who knew the individual's identity. Form-filling was not verified or supervised because that would have nullified the anonymity, but relied on the pilot trial to have eliminated ambiguities in the description of what was required.

The stalkers collected the data on which this research is based as an incidental add-on to routine deer control carried out as part of deer population management on private land; no deer was killed specifically in pursuit of the research objective. Owners and occupiers of land in the UK have the legal right to kill deer on their land under the Deer Act 1991 and the Deer (Scotland) Act 1996, and can lawfully delegate that right to others. In this study, all stalkers had permission to shoot deer from the owners of the land or those with the right to kill deer there. The stalkers held legal Police Firearms Certificates, used legal firearms and ammunition as authorised by the Deer Acts, culled deer when they were in season and complied with the Code of Practice for deer stalking [25]. Because the animals were being killed as part of standard wildlife management, the data collection did not require a licence under the Animals (Scientific Procedures) Act 1986, or any government oversight or approval. The project was approved by the Scientific/Research and Training Committees of the British Deer Society, and passed scrutiny by the Local Ethical Review Committee of the Game & Wildlife Conservation Trust.

### Preparation of data for analysis

During data entry, checks were carried out to verify that all variable codes were consistent with the instructions issued to participants, and that records could be matched up across forms. Records with inconsistencies or that could not be matched up were omitted from the analysis. We also excluded the single shot that had been fired from a shotgun (to kill a sick roe deer at close range); all other shots were from rifles. Only four shots had been fired at Chinese water deer and these were also excluded from the analysis, as was a single shot at an unrecorded species of deer.

With regard to the outcome of each shot (Form S2), an animal was classed as killed if it was hit by the shot and died (no blink reaction when an eyeball was touched) within two minutes. It was classed as hit and wounded if the animal required another shot to kill it, as hit and lost if it was wounded but not fired at again (animal escaped), or as a clean miss if the shot did not hit the target (no behavioural or physical indicators of a hit such as the animal's physical reaction to the shot and any signs of blood or hair left where it had been standing). In practice, the difference between wounded and killed on the first shot was not always clear-cut. For instance, a heart-shot deer may technically “live” for up to two minutes before brain death but is effectively killed. Where this was specified in the stalker's comments, the deer was treated as being killed with the first shot. In other cases of multiple shots at the same deer, accompanying comments from the stalkers indicated that the circumstances surrounding second and any subsequent shots were rather different to the first shot and much more variable. To ensure comparability, only first shots were retained for analysis, so that each shot corresponded to a different animal and gave rise to a single record in the dataset.

We set up two binary variables to represent the outcome of each shot, and serve as response variables in the analysis. The first variable represented the probability of hitting a target deer, and was coded 1 if the shot hit, 0 if it missed. The second variable represented the probability that the shot killed the deer if the animal was hit: it was coded 1 if the shot killed, 0 if it wounded, and was not applicable if the shot missed.

From the data forms and stalker register, we derived 28 potential explanatory variables that, for each shot, provided information on the stalker, the targeted deer and the circumstances of the shot (Table 1). Twenty-five of these variables were recorded as categorical in the field or from the stalker register. For analysis, we sought to balance the number of categories with the need for reasonable sample sizes when considering combinations of variables. We therefore eliminated categories with small sample sizes by combining them with other categories. Distance to target, called ‘range’ on Form S1, was a continuous variable recorded in the field as estimated and measured distances (to the nearest metre). The two measurements were found to be very close (r^2^ = 0.95, n = 2011). Estimated distance was retained as the measurement of distance to target because it had the fewest missing values; these were further reduced by substituting measured distance values if they existed. It was then categorised as <75 m, 75–149 m, 150+ m (based on stalker perception of relevant distances) to check for linearity of a relationship during analysis. Bullet weight was also recorded as a continuous variable (to the nearest grain), and divided into 25-grain categories for the same reason. Muzzle energy (E) in Joules was derived from bullet weight via the equation E = mv^2^/2, where m =  bullet weight in grams and v =  muzzle velocity in metres per second (according to firearm manufacturer specifications). It was subdivided into 500-J categories for analysis. For these three variables, if during analysis the pattern of change across categories was found to be linear, the original continuous variable was retained.

**Table 1:** Variables and their categories describing the circumstances of 2281 first shots fired at deer in the UK, together with information on the stalker and the targeted deer.

Variable	Categorisation	Number of categories	Values present per category	Values missing
Stalker age	<40, 40–49, 50–59 and 60+ years	4	354, 874, 755, 298	0
Years of experience	<5, 5–14, 15+ years of stalking experience	3	410, 729, 1142	0
Deer shot per year	<10, 10–24, 25+ animals (based on 1999–2003 average, all species combined)	3	529, 784, 968	0
Qualification	None, Deer Stalking Certificate 1, Deer Stalking Certificate 2, Advanced Stalker Certificate	4	378, 814, 796, 293	0
Zero check	Under once a year, 1–12 times a year, over once a month, over once a week	4	43, 1508, 572, 151	7
Shooting practice	Under once a year, 1–12 times a year, over once a month, over once a week	4	189, 1409, 484, 178	21
Rifle calibre	.22 (.222,.22–243,.22–250),.24–.26 (.240,.243,.260, 6 mm, 6.5 mm),.27–.29 (.25–06,.270,.275, 7 mm),.30–.34 (.300,.308,.30–06,.338)	4	43, 1051, 606, 581	0
Bullet weight	<75, 75–99, 100–124, 125–149, 150+ grains	5	60, 250, 964, 503, 504	0
Muzzle energy	<2000, 2000–2499, 2500–2999, 3000–3499, 3500–3999, 4000–4999, 5000+ Joules	7	58, 239, 907, 544, 485, 27, 21	0
Shooting position	Lying/prone, sitting/kneeling/crouching, standing, high seat/vehicle	4	765, 330, 767, 417	2
Use of gun rest	Flat rest, bipod, sticks, high seat rail, vertical rest, off elbows, freehand	7	301, 596, 602, 349, 227, 131, 69	6
Comfort	Comfortable/steady, uncomfortable/unsteady	2	1806, 228	247
Time available for shot	Hurried, very little time, sufficient time, more than adequate time	4	404, 631, 819, 424	3
Point of aim	Chest, low neck, high neck, head	4	1688, 193, 247, 142	11
Distance to target	<75, 75–149, 150+ metres	3	958, 983, 336	4
Light	Very bright, quite bright, dull, quite dark, just legal, night shooting	6	455, 797, 717, 218, 73, 14	7
Weather	Fine, rain, hail/snow, fog/mist	4	1533, 206, 37, 51	454
Wind strength	0–10, 11–20, 21–30, 31–40 miles per hour	4	1590, 455, 78, 24	134
Wind angle	Head wind (11, 12, 1 o'clock), from left (2, 3, 4 o'clock), from behind (5, 6, 7 o'clock), from right (8, 9, 10 o'clock), no wind	5	576, 594, 187, 671, 42	211
Known area	Stalked often, stalked regularly, stalked occasionally, not well known, new area	5	1207, 462, 279, 140, 186	7
Habitat type	Open ground, fields, small woods/fields, open woodland, thick woodland, bare hillside	6	324, 573, 391, 491, 275, 226	1
Ground vegetation	Bare ground, height <0.5 m, height <1 m, height 1–2 m, woodland, thicket	6	630, 1010, 330, 114, 144, 45	8
Concealment	Unobscured, partially obscured, heavily obscured	3	1487, 473, 79	242
Deer species	Red, sika, fallow, roe, muntjac	5	355, 183, 409, 1179, 155	0
Deer sex	Stag/buck, hind/doe	2	1121, 1157	3
Deer age	Under 1 year, 1–2 years, mature adult, old	4	474, 701, 919, 161	26
Alone or group	Alone, small group (2–4), large group (5–10), herd (10+)	4	819, 1040, 263, 151	8
Alert state	Unaware of stalker, alert, suspicious, looking intently at stalker, about to run, on the move	6	983, 295, 371, 304, 241, 87	0
Deer orientation	Facing away (11, 12, 1 o'clock), facing right (2, 3, 4 o'clock), facing left (8, 9, 10 o'clock), head-on (5, 6, 7 o'clock)	4	133, 944, 902, 298	4

For each variable, the sample size for each category (number of shots with data present) and the number of missing values are also given.

Because stalkers did not always complete every field on the forms, there were missing values in many of the variables (Table 1). Overall, only 1325 data records out of 2281 contained no missing values (Dataset S1). This meant that for a joint analysis involving all explanatory variables, an approach that considered only records with no missing values would reject 42% of the dataset. We considered this extent of data loss to be unacceptable, so we replaced missing values for a given variable using non-missing values from other records chosen at random from a list of suitable matches (‘hot-deck imputation’ [26]) using the HOTDECK procedure in Genstat 16th Edition [27]. The matches were determined on the basis of similarity to potential donor records based on a set of reference variables – we used stalker variables if imputation was for a missing stalker characteristic, deer variables if imputation was for a deer characteristic and shot variables if the imputation was for a variable describing the circumstances of a shot. The best match was taken to be the one with the minimum value of the maximum absolute difference (standardised for range) between any of the reference variables. To prevent the same donor being selected repeatedly, the actual match was selected at random from amongst potential donors with distance values up to 10% greater than the minimum distance. To avoid undue influence of a particular random configuration of the imputed dataset, we applied hot-deck imputation ten times to generate ten completed datasets.

### Statistical analysis

We fitted generalised linear mixed models (GLMMs) to the probability that a first shot hit a target deer, and to the probability that the shot killed the deer if it was hit. We modelled these two variables separately as Bernouilli trials (binomial error, logistic link, binomial denominator equal to one). Because most stalkers fired shots at more than one animal (range 1–72, average 22 shots per stalker), we included stalker ID as a random effect in the GLMMs. We tested for the effect of explanatory variables included as fixed effects using Wald tests [28]. Statistical modelling was carried out in Genstat 16th Edition [27], and involved an automated internal conversion of categorical explanatory variables to dummy variables coded 0 or 1 [29].

We first tested for species-specific differences in the probabilities of hitting and killing a target animal by including species as a fixed explanatory factor in the model. Then, considering each potential explanatory variable in turn from Table 1, we checked for statistical interactions between species and the explanatory variable. In the presence of such interactions, it would have been necessary to consider each species separately, leading to much reduced sample sizes. In the event, no significant (P<0.05) interactions were detected (Table S1), so data were analysed across all species at once.

We then sought to identify which potential explanatory variables from Table 1 were most closely associated with each of the response variables. Variable selection in the case of mixed models is a developing area [30], and some statisticians consider the use of Akaike's Information Criterion and related techniques invalid for fixed effects (e.g. [28]). An attractive new technique is the lasso [31], a shrinkage method that avoids comparing large numbers of models. The technique has been expanded to generalised mixed models and categorical variables by Schelldorfer et al. [32], but the algorithms would not converge for our dataset. We therefore adopted traditional stepwise selection based on Wald tests, whereby each potential explanatory variable in turn was considered as a fixed effect in a statistical model that started with species as a factor. The model was gradually built up with, in turn, each of the remaining variables that explained the greatest amount of variation in the data while being significant at P<0.05. A variable that became non-significant as the model expanded was removed from the model. Draper & Smith [29] consider this stepwise selection approach to be superior to a backwards elimination approach. The variance inflation factors (VIFs) for the explanatory variables were well below 10 [33] except for Rifle calibre, Bullet weight and Muzzle energy (Table S2), where each variable caused high VIFs in one of the other two. In practice this was not an issue because only one of the three was ever selected at a time.

Selection consistency across the ten completed datasets was achieved by appending them one after the other for a joint analysis (Dataset S2) and fitting GLMMs with common parameter estimates for fixed effects [34], [35]. Datasets remained individually identifiable through a categorical dataset variable coded 1–10, which was used to keep random effects and constants dataset-specific. Hence the starting model contained stalker ID within dataset as a random effect, and dataset together with species as fixed effects. The stepwise procedure then added further explanatory variables (Table 1) to the fixed effects. Wald statistics testing for significance of fixed effects were divided by 10 to correct for the ten-fold replication. To aid the interpretation of effects in the final models, pairwise comparisons between the different categories in each of the selected explanatory variables were carried out using Wald tests with P = 0.05 as the nominal level for significance.

## Results

### Outcome of shots

In total, 102 stalkers fired 2281 first shots at deer, resulting in:

− Clean misses: 102 (4.5%)

− Hits: 2179 (95.5%)

The deer that were hit divided into:

− Killed outright: 2026 (88.8%)

− Wounded: 153 (6.7%)

The wounded deer were subdivided into:

− Killed with subsequent shot: 125 (5.5%)

− Lost/escaped: 28 (1.2%)

Based on the numbers above, 93.0% of first shots that hit the target animal resulted in an outright kill, and 81.7% of wounded animals were killed with a subsequent shot.

### Differences between deer species

After adjusting for stalker effects, the estimated probability of a shot hitting its target did not differ between species (Table 2). In addition, none of the interactions between species and the other explanatory variables in Table 1 was significant (Table S1).

**Table 2:** Shooting accuracy for deer in the UK: probability of the first shot hitting the target, and of killing the animal when it was hit, by target species.

Deer species	Probability of hit from first shot			Probability of kill when first shot hit		
	Number of shots	Probability estimate (%)	95% confidence interval	Number of shots	Probability estimate (%)	95% confidence interval
Red	355	94.5	91.4–96.6	336	88.4	84.2–91.6
Sika	183	96.8	92.8–98.7	177	90.2	83.6–94.3
Fallow	409	97.2	95.0–98.4	397	92.4	89.0–94.8
Roe	1179	94.7	93.2–96.0	1117	94.3	92.7–95.6
Muntjac	155	98.1	94.2–99.4	152	99.3	95.4–99.9
Overall	2281	95.5	91.5–97.7	2179	93.0	91.6–94.2
						
Wald		7.90, 4 df, P = 0.095			23.12, 4 df, P<0.001	

Wald statistics test for overall differences between species.

If a shot hit its target, the probability that it killed the animal varied between species from 88% for red deer to 99% for muntjac (Table 2). None of the interactions between species and the other explanatory variables was significant (Table S1).

### Factors associated with hitting or missing a deer

**Figure 1:**
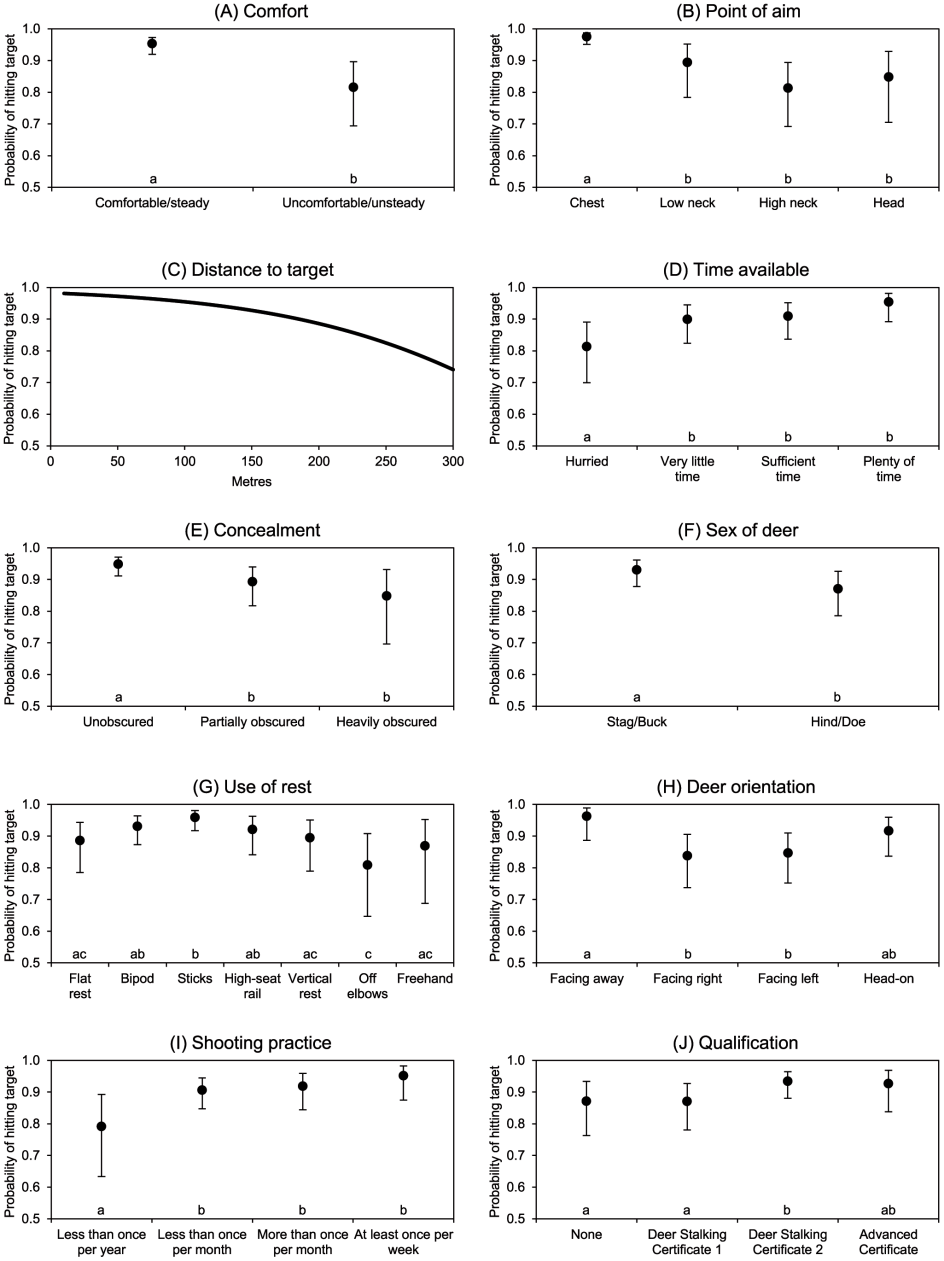
Probability that a shot hit the target deer, in relation to ten variables. The variables were identified as significant (Table 3) using stepwise selection within a generalised linear mixed modelling framework: (A) Comfort of firing position, (B) Point of aim, (C) Distance to target, (D) Time available for the shot, (E) Concealment of target, (F) Sex of target deer, (G) Use of gun rest, (H) Deer orientation, (I) Frequency of shooting practice, (J) Stalker qualification. In each graph, the probabilities are adjusted for the effects of the other selected variables. The error bars represent 95% confidence limits. For each categorical variable, categories with the same letter do not differ at P<0.05.

Ten variables were identified as being significantly associated with the probability of a shot hitting its target (Table 3; Figure 1). None of the two-way interactions among these variables was significant. The nature of the associations between the probability of a shot hitting its target and each of these variables (in order of selection) is examined below.

**Table 3:** Factors associated with shooting accuracy: variables from Table 1 significantly associated with the probability of a first shot hitting a target deer, and of killing an animal when it was hit, presented in the order of stepwise selection within a generalised linear mixed modelling framework (see Methods).

Selection order	Probability of hit from first shot				Probability of kill when first shot hit			
	Variable	Wald	df	P	Variable	Wald	df	P
1	Comfort	34.18	1	<0.001	Comfort	20.09	1	<0.001
2	Point of aim	47.63	3	<0.001	Distance to target	16.65	1	<0.001
3	Distance to target	23.77	1	<0.001	Time available	12.51	3	0.006
4	Time available	14.84	3	0.002	Bullet weight	15.51	4	0.005
5	Concealment	14.73	2	0.001	Alert state	16.37	5	0.006
6	Deer sex	8.61	1	0.003	Known area	12.72	4	0.013
7	Use of rest	18.95	6	0.004	Ground vegetation	12.95	5	0.024
8	Deer orientation	9.50	3	0.023	Distance x Time	11.31	3	0.010
9	Shooting practice	10.57	3	0.014				
10	Qualification	9.01	3	0.029				

**Figure 2:**
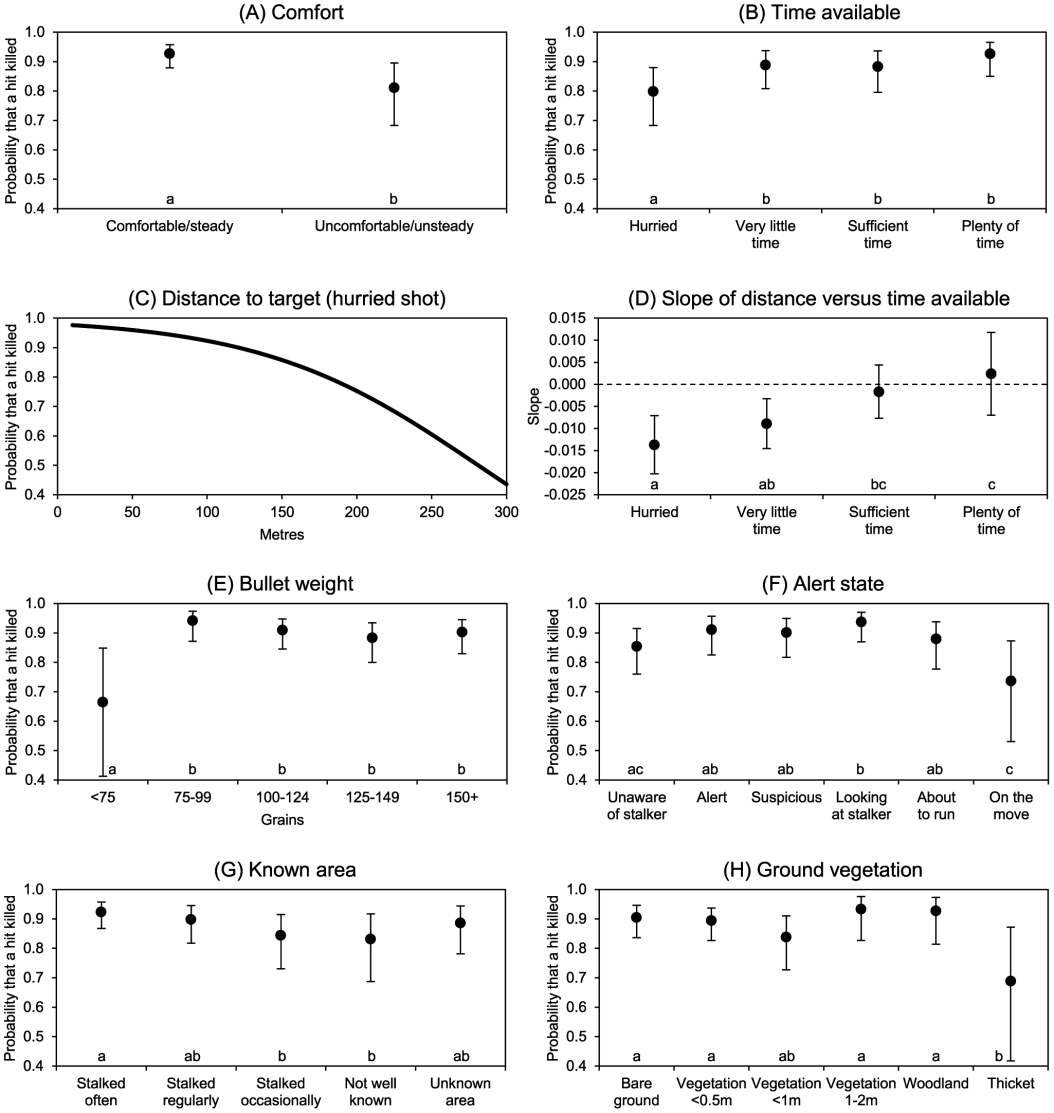
Probability that a shot that hit killed its target, in relation to seven variables. The variables were identified as significant (Table 3) using stepwise selection within a generalised linear mixed modelling framework: (A) Comfort of firing position, (B) Time available for the shot, (C) Distance to target (when shot is hurried), (D) Slope of relationship with Distance to target in relation to Time available (on logistic scale), (E) Bullet weight, (F) Deer alert state, (G) Knowledge of area, (H) Ground vegetation. In each graph, the probabilities are adjusted for the effects of the other selected variables. The error bars represent 95% confidence limits. For each categorical variable, categories with the same letter do not differ at P<0.05.

Wald statistics and levels of significance are taken from the model containing all selected variables. The directionality and magnitude of effects are shown in Figures 1 and 2.

Comfort (Figure 1A). The comfort and stability of the firing position was the variable most strongly associated with the probability of a shot hitting its target. It varied between 95% for a comfortable (stable) firing position and 82% for an uncomfortable (unstable) one.

Point of aim (Figure 1B). This was also highly associated with the probability of a shot hitting its target. Shots at the chest were most likely to hit (97% success), followed by shots to the lower neck (89%). Shots to the high neck were least likely to hit (81%) and ones to the head were intermediate (85%).

Distance to target (Figure 1C). The probability of a shot hitting its target declined increasingly with distance, from 95% (distances under 75 m) to 91% (distances of 75–149 m) then 83% (distances of 150+ m). The relationship was linear on the logistic scale, and when distance was considered as a continuous variable the slope was −0.010, giving the relationship P(hit) = 57/(57+e^0.010 distance^). The relationship described by this equation is shown graphically in Figure 1C.

Time available (Figure 1D). The probability of a shot hitting its target increased with the amount of time available. It was lowest when the shot was hurried (81%), and highest with more than enough time (95%).

Concealment (Figure 1E). The probability of a shot hitting its target decreased as the degree of concealment increased, from 95% when the target animal was unobscured, down to 85% when the target animal was heavily obscured.

Sex (Figure 1F). Stags and bucks were more likely to be hit (success rate of 91%) than hinds and does (85%).

Use of rest (Figure 1G). Of the types of gun rest used, sticks were associated with the highest success rate (96%), followed by bipods (93%) and high-seat rails (92%). Flat rests and vertical rests were very similar to each other, at 89%. Freehand and shooting off elbows were the least successful methods (87% and 81% respectively).

Deer orientation (Figure 1H). The probability of a shot hitting its target was highest for animals facing away from the stalker (96%), and lowest for animals facing right or left (84–85%). It was 92% for head-on animals.

Shooting practice (Figure 1I). The probability of a shot hitting its target was highest for stalkers who practised at least once a week (95%). It fell slightly, to 91–92%, with some annual practice, then dropped to 79% with practice less than once a year.

Qualification (Figure 1J). The probability of a shot hitting its target was highest for stalkers with a Deer Stalking Certificate 2 or an Advanced Stalker Certificate (93%). It was lower for stalkers with a Deer Stalking Certificate 1 or no qualification, at 87%.

The results for Deer orientation seemed counter-intuitive because a broadside target is larger than one facing away or head-on, so they were examined more closely. The selection of Deer orientation depended strongly on the presence of the Point of aim variable in the model (if Point of aim was omitted, the level of significance of Deer orientation dropped to P = 0.849). The association between Deer orientation and Point of aim is displayed in Table 4, showing that a high majority of shots were broadside shots to the chest (81% of 2267 shots were to deer facing right or left, and 84% of those were to the chest). Within our sample, 100% of shots to the chest when deer were facing away or head-on hit the target, whereas 96% of broadside shots to the chest hit the target. This pattern, combined with the large overall sample size for shots to the chest, produced the probabilities observed for Deer orientation after adjusting for Point of aim. Taking both factors into account (Table 4), the observed success rate for broadside shots to the chest was 96%.

**Table 4:** Percentage (sample size in brackets) of first shots that hit the target in relation to point of aim and deer orientation (2267 shots, after excluding 14 with missing information).

Deer orientation	Point of aim			
	Chest	Low neck	High neck	Head
Facing away	100.0 (40)	100.0 (18)	90.5 (42)	100.0 (30)
Facing right	96.2 (786)	96.9 (64)	85.9 (64)	88.9 (27)
Facing left	96.3 (755)	89.1 (55)	94.8 (58)	87.9 (33)
Head-on	100.0 (106)	92.9 (56)	92.6 (81)	94.2 (52)

### Factors associated with killing or wounding a deer that has been hit

Seven variables were identified as being significantly associated with the probability of a shot killing the animal that it hit (Table 3; Figure 2). There was a significant two-way interaction between Distance to target and Time available, which we describe below under Distance to target. In order of selection:

Comfort (Figure 2A). As before, the comfort and steadiness of the firing position was the factor most strongly associated with a straight kill. It varied from 93% for a comfortable (stable) firing position to 81% for an uncomfortable (unstable) one.

Time available (Figure 2B). The probability of a hit animal being killed was lowest in the case of a hurried shot (80%), and highest when there was plenty of time (93%).

Distance to target (Figure 2C, 2D). The statistical interaction between distance and time available arose because there was no detectable effect of distance on the probability of a hit animal being killed when there was sufficient or more than enough time (Figure 2D; slopes not significantly different from zero, implying that the probability did not change with distance). However, distance grew in importance when there was very little time (slope of −0.009) and more so when the shot was hurried (slope of −0.014). Figure 2C shows how the probability of a hit animal being killed declined with distance in the latter case (equation P(kill|hit) = 47/(47+e^0.014 distance^); the relationship was similar, but less pronounced, when there was very little time.

Bullet weight (Figure 2E). The probability of a hit animal being killed was highest for bullets in the 75–99 grain range (94%), followed by those in the heavier weight categories (88–91%). Bullets weighing less than 75 grains performed markedly less well (probability of 66%). The large 95% confidence interval for this category was because of a small sample size of 54 shots.

Alert state (Figure 2F). The probability of a hit animal being killed was lowest when the animal was on the move (74%) or unaware of the observer (85%). It was highest for the situations where an animal was stationary and alert, suspicious or looking at the stalker (90–93%). The large 95% confidence interval for the category of animals on the move was because of a small sample size of 78 shots.

Known area (Figure 2G). The highest probabilities of a hit animal being killed were associated with ground that was stalked often or regularly (90–92%). The lowest probabilities were associated with ground that was stalked occasionally or was not well known (83–84%), while they were intermediate on ground that was unknown (88%).

Ground vegetation (Figure 2H). The probability that a hit animal would be killed was lowest for thicket (69%). It was highest for vegetation 1–2 m in height and woodland vegetation (93%), followed by bare ground and ground with vegetation under 0.5 m tall (89–90%). The large 95% confidence interval for thicket was because of a small sample size of 42 shots.

For Bullet weight, the poor performance of bullets under 75 grains warranted further investigation to establish whether bullet weight or rifle calibre was driving the result. The original data showed that 54 shots from five stalkers involved bullets weighing less than 75 grains. Two of the shots were fired from a high-velocity Remington 22–250 rifle, and both shots killed their targets. The remaining 52 shots were fired from rifles of calibre.222,.243 or 6 mm, with muzzle energy below 2000 J. All were fired at roe deer and 17.3% of these shots resulted in wounding. There was no obvious effect of distance, as only one of the wounding shots extended beyond 100 m. Three of the same stalkers also fired 22 shots at roe deer from rifles of identical calibre using bullets weighing over 75 grains (muzzle energy above 2500 J): there was no incidence of wounding. For comparison, another 37 stalkers fired 440 shots at roe deer from rifles of identical calibre using bullets over 75 grains: the wounding rate was 4.5%.

## Discussion

Deer management in the UK brings with it a responsibility for maximising deer welfare, which in this case equates to killing the quarry speedily and with a minimum of suffering [20]. The ideal is to kill the quarry with a single shot; this study has quantified to what extent this ideal is achieved in practice by estimating the probabilities of a shot being successful, both in terms of it hitting the target animal, and of it killing rather than wounding the animal when it has hit its target.

We found that the probability of hitting a deer varied relatively little between deer species (95–98%), whereas the probability of killing a hit animal ranged more widely from 88% to 99% (Table 2), giving estimated wounding rates of 1–12%. When a deer was not killed outright by the first shot and then escaped, determining whether it was hit or not was done using behavioural and physical indicators such as the animal's reaction to the shot and any blood or hair signs left where it had been standing. There was a possibility that, if such signs were not noticed or discovered, a deer could have been reported as a clean miss when in fact it had been hit. The worst-case scenario of all animals not killed being wounded gives upper limits to the wounding rates of 3–17% (Table 5).

**Table 5:** Maximum wounding rates: upper limits to the percentage of deer that were wounded after being shot at, by species.

Deer species	Miss rate (%)^1^	Wounding rate (%)^2^	Wounding rate upper limit (%)^3^
Red	5.5	11.6	16.5
Sika	3.2	9.8	12.7
Fallow	2.8	7.6	10.2
Roe	5.3	5.7	10.7
Muntjac	1.9	0.7	2.6

1 From third column of Table 2.

2 From sixth column of Table 2.

3 Calculated as 1 - (1 - miss rate) * (1 - wounding rate).

To put these wounding rates in context, Bateson & Bradshaw [23] reported a wounding rate of 10% for red deer based on carcasses at game dealers, while Bradshaw & Bateson [36] used estimates of wounding rates by stalkers to show that 11% of red deer required two or more shots to kill in southwest England. Urquhart & McKendrick [24] found that 14% of red deer carcasses from Scottish stalkers had more than one wound tract. These rates are similar to the 12% obtained in this study directly from the stalkers. All the wounding rate estimates from this study, including all of their upper 95% confidence limits, were lower than those reported in a study of hunter-inflicted wounding of White-tailed Deer *Odocoileus virginianus* in Indiana, which ranged between 17% and 32% [37].

In terms of welfare, the obvious question arising from these figures is what factors affect the probability of wounding, because a good understanding of them may allow them to be manipulated for the better. This study sought to explain the variation in the probabilities of a shot hitting a target deer, and of killing a deer that was hit. The most important one for deer welfare is the second, because if a deer was not killed it was wounded and hence was suffering. Our statistical modelling of this second probability allowed us to identify the factors directly associated with a high chance of an outright kill – the most humane outcome – and those associated with a high probability of wounding, which should be avoided. However, we cannot ignore the first probability because, as discussed earlier, we cannot exclude the possibility that a deer could be reported as a clean miss when in fact it had been hit and wounded. Moreover, it is clearly more efficient for stalkers engaged in deer management not to miss the targets that they are aiming at. It is therefore valuable also to consider what factors maximise the probability of hitting a target animal.

The two probabilities, that a shot hit a target deer, and that it killed a deer that was hit, were associated with a set of ten and seven variables respectively that explained significant amounts of variation. Three variables were common to both sets, namely the comfort of the shooting position, the distance to the target and the time available for the shot. A further similarity existed between concealment (first set) and ground vegetation (second set), which were strongly inter-related. The two response probabilities varied in the same way with respect to comfort, distance to target, time available and concealment/vegetation. Thus an uncomfortable shooting position was associated with lower probabilities (more misses and more wounding) than a comfortable one, both probabilities generally decreased with distance to the target (for the probability that a shot killed a deer that was hit, only when the shot was rushed) and when the target was heavily obscured/in thicket. These findings are in accordance with what we would expect.

For the probability that a shot hit a target deer, the six remaining selected variables were point of aim, use of rest, sex of the deer, deer orientation, shooting practice and stalker qualification. For point of aim, the probability of a hit was highest for shots aimed at the chest, which gives the greatest margin for error, and lowest for shots to small target areas such as the head and high neck. The probability of a hit was reduced when deer were broadside on – perhaps because such a target is seen as “easy” and less care is taken - but the result has no practical implication because the point of aim was closely associated with the orientation of the deer. Shots were taken to the chest in the vast majority of cases (84%) when the deer was facing left or right, and the observed percentage of hits in this situation was high at 96%. As regards the use of rifle rests, those associated with the highest probability of hitting the target were sticks and bipods, while shooting off elbows and freehand shooting, where the rifle barrel is not firmly supported, led to the lowest probabilities among rests in this study. The probability of hitting the target was found to be higher for male deer than for females, which may be a function of timing (females are stalked in winter, males often outside winter), quantity (stalkers often try for two does but only one male at a time) or size (males are often larger than females).

The last two variables described stalker qualities. The probability of hitting a target deer was high with frequent shooting practice, but dropped considerably with a lack of it. Likewise, Deer Stalking Certificate 2 and Advanced Stalker Certificate holders had a higher probability of hitting a target animal than stalkers with a Deer Stalking Certificate 1 or no qualification. This perhaps reflected nervousness or excitement at the moment of squeezing the trigger, or a lack of skill in holding a rifle steady when firing, on the part of less experienced stalkers. By comparison, Deer Stalking Certificate 2 and Advanced Stalker Certificate holders enjoyed greater experience and had shot higher numbers of deer per year. Taken together, these results suggest that training for Deer Stalking Certificate 1 should include additional firing practice under field conditions, and that stalkers should undertake target practice at least once a month.

With regard to the probability of a shot killing a deer that had been hit, which was directly related to the incidence of wounding, the weight of the bullet appeared important. The probability of a kill was particularly low, and wounding correspondingly high, for bullets weighing less than 75 grains. The energy of the shot was closely related to bullet weight, and we found that energy was under 2000 J for bullets under 75 grains fired at roe deer in this study. Such bullets are legal for roe deer in Scotland (Deer (Firearms etc.) (Scotland) Order 1985), where bullets above 50 grains and 1356 J are permitted, but are under the legal limit of 2305 J in England and Wales (Deer Act 1991). Given that wounding rates with bullets weighing less than 75 grains were four times higher than wounding rates with heavier bullets in identical-calibre rifles, it seems that the issue lies with bullet weight/energy and not calibre. The results obtained here suggest that the English and Welsh limit provides a better safeguard against excessive wounding than the Scottish one. Because of the low sample sizes involved (only five stalkers used the light bullets), it would be sensible to set up an experimental trial to test this suggestion.

Other variables associated with the probability of killing a hit animal were the alert state of the deer and the stalker's knowledge of the area. Shots at moving targets were associated with more wounding than shots at deer that were alert, suspicious or looking at the stalker, which were most likely to have been stationary (although the shot may have been more hurried than would otherwise have been the case). The probability of a clean kill was intermediate for deer unaware of the stalker, possibly because such deer may have been grazing or browsing and hence slowly moving, and for deer about to run, which may have just started moving as the shot went off. Finally, how well a stalker knew the ground also appeared to play a role, with the likelihood of a clean kill associated with ground stalked often or regularly.

At no stage of the analyses were any of the weather variables selected. Thus rain, snow, fog or wind appeared to be unimportant in determining the probability of hitting an animal or of killing it if it was hit. This may simply be because stalkers avoid weather so bad that it would affect their shooting, or it may be that they are genuinely unimportant relative to the other factors that have been identified.

This study has therefore identiifed a range of factors that affect the probability that a shot deer is wounded rather than killed, with obvious welfare consequences. In essence, the more complicated or challenging the environment is for shooting deer, the more the probability of their welfare being compromised increases. Stalkers may have to make compromises to achieve the best possible welfare outcome. For instance, when selecting a point of aim, a shot that destroys the brain achieves the ideal outcome of an outright kill, but leaves very little room for marksman error. A shot that is even slightly misplaced (owing perhaps to forces outside stalker control such as a gust of wind or a slight movement of the deer's head) can result in a broken jaw and, if the animal escapes, the worst possible outcome [38]. Overall, better deer welfare is achieved with a hit in the chest (a larger more stable target), which together with a legally mandatory expanding bullet causes major damage to the deer's vital organs (heart/lungs/liver) and massive blood loss followed by unconciousness and rapid death [39]. The conditions that minimise the chances of wounding are those that should be enshrined as best practice. In the conclusions that follow, we list the criteria that this study indicates should form part of best practice.

## Conclusions

Shooting deer for sport or population control brings with it a welfare responsibility to minimise stress and suffering, best achieved by an outright kill with the first shot. The results of this study largely confirm previous best practice teaching, but also offer several clear pointers for improving welfare by maximising the chances of an quick and certain kill and minimising wounding. These are summed up as follows:

- Choose a comfortable shooting position.
- Avoid shooting off elbows or freehand, use a support (e.g. sticks, bipod).
- Aim at the chest or lower neck.
- Use bullets heavier than 75 grains.
- Avoid shooting when there is insufficient time.
- Shoot a distant animal only if there is sufficient time.
- Shoot an animal when it is stationary.
- Avoid shooting an animal that is heavily obscured or in thicket.
- Take extra care when the ground is not well known.

(j) Carry out shooting practice at least once a month.

In addition, the relatively high rate of missed shots by Level 1 stalkers suggests that training for a Level 1 qualification should include additional firing practice under realistic field conditions (less than comfortable/little time) and perhaps further training in ensuring that any hit is reliably detected.

The intention is to bring these results to the attention of trainee and experienced stalkers by incorporating them into training courses, guidance notes and other information outlets. Increasing awareness in these ways should deliver improvements in shooting standards that ensure that when stalkers take a shot, they are killing the animal in a way that minimises suffering, thereby maximising deer welfare.

## Supporting Information

Table S1 Tests for interactions between species and other explanatory variables. Statistical significance (based on Wald statistics) of the interactions between deer species and the explanatory variables in Table 1 when modelling the probability that a shot hit its target, and the probability that a shot that hit its target killed the animal.
(PDF)

Table S2 Variance inflation factors for explanatory variables. Variance inflation factors (VIFs) for the explanatory variables in Table 1 when modelling the probability that a shot hit its target, and the probability that a shot that hit its target killed the animal. Categorical variables have one VIF per category, so the minimum and maximum VIFs are given to show the range.
(PDF)

Dataset S1 Field records of first shots at deer collected from stalkers.
(XLSX)

Dataset S2 Ten hotdecked datasets appended together for the stepwise selection analysis.
(XLSX)

Form S1 Instructions and form for collecting data on shots fired at deer.
(PDF)

Form S2 Instructions and form for collecting data on deer fired at.
(PDF)
