# Supplementary material for: Factors Associated with Shooting Accuracy and Wounding Rate of Four Managed Wild Deer Species in the UK, Based on Anonymous Field Records from Deer Stalkers
Source: PLoS One. 2014 Oct 15;9(10):e109698. doi: 10.1371/journal.pone.0109698 (PMC4198128; doi:10.1371/journal.pone.0109698)
Supplement: Table S1 — Tests for interactions between species and other explanatory variables. Statistical significance (based on Wald statistics) of the interactions between deer species and the explanatory variables in Table 1 when modelling the probability that a shot hit its target, and the probability that a shot that hit its target killed the animal. (PDF) [file pone.0109698.s001.pdf]

Table S1. Statistical significance (based on Wald statistics) of the interactions between deer species and the explanatory variables in Table 1 when modelling the probability that a shot hit its target, and the probability that a shot that hit its target killed the animal.

**Variable**	**Probability of hit from first shot**			**Probability of kill when first shot hit**		
	**Wald**	**df**	**P**	**Wald**	**df**	**P**
Stalker age	7.43	11	0.763	2.18	11	0.998
Years of experience	4.06	8	0.852	3.64	8	0.888
Deer shot per year	6.42	8	0.600	6.50	8	0.591
Qualification	6.58	12	0.884	7.65	12	0.812
Zero check	2.60	10	0.989	1.00	10	1.000
Shooting practice	4.09	11	0.967	3.27	11	0.987
Rifle calibre	1.64	9	0.996	2.45	9	0.982
Bullet weight	0.24	14	0.999	3.89	14	0.996
Muzzle energy	1.86	17	0.999	7.75	16	0.956
Shooting position	5.85	12	0.924	9.66	12	0.646
Use of rest	10.16	24	0.994	13.57	24	0.956
Comfort	2.69	4	0.611	0.97	4	0.915
Time available	4.71	12	0.967	8.09	12	0.778
Point of aim	6.49	12	0.889	13.41	12	0.340
Distance to target	4.95	4	0.292	2.24	4	0.691
Light	7.44	18	0.986	11.45	17	0.832
Weather	2.48	12	0.998	8.49	12	0.745

Wind strength	2.54	12	0.998	5.41	12	0.943
Wind angle	2.34	17	0.999	8.40	16	0.936
Known area	9.22	17	0.933	5.76	16	0.990
Habitat type	5.99	20	0.999	12.53	20	0.897
Ground vegetation	5.48	20	0.999	5.82	20	0.999
Concealment	2.06	8	0.979	7.19	8	0.517
Deer sex	7.63	4	0.106	4.47	4	0.347
Deer age	5.31	12	0.947	8.86	12	0.715
Alone or group	4.96	10	0.894	14.04	10	0.171
Alert state	15.78	20	0.730	15.06	20	0.773
Deer orientation	8.59	12	0.737	9.67	12	0.644
