# Supplementary material for: Factors Associated with Shooting Accuracy and Wounding Rate of Four Managed Wild Deer Species in the UK, Based on Anonymous Field Records from Deer Stalkers
Source: PLoS One. 2014 Oct 15;9(10):e109698. doi: 10.1371/journal.pone.0109698 (PMC4198128; doi:10.1371/journal.pone.0109698)
Supplement: Table S2 — Variance inflation factors for explanatory variables. Variance inflation factors (VIFs) for the explanatory variables in Table 1 when modelling the probability that a shot hit its target, and the probability that a shot that hit its target killed the animal. Categorical variables have one VIF per category, so the minimum and maximum VIFs are given to show the range. (PDF) [file pone.0109698.s002.pdf]

Table S2. Variance inflation factors (VIFs) for the explanatory variables in Table 1 when modelling the probability that a shot hit its target, and the probability that a shot that hit its target killed the animal. Categorical variables have one VIF per category, so the minimum and maximum VIFs are given to show the range.

**Variable**	**Probability of hit from first shot**	**Probability of kill when first shot hit**
Stalker age	1.80-2.23	1.81-2.22
Years of experience	2.15-2.57	2.16-2.59
Deer shot per year	1.85-2.50	1.82-2.53
Qualification	3.86-5.24	3.83-5.16
Zero check	1.49-2.59	1.51-2.60
Shooting practice	1.83-2.20	1.81-2.21
Rifle calibre	24.79-27.59	25.34-28.22
Bullet weight	2.12-34.73	2.13-31.57
Muzzle energy	1.72-32.00	1.73-28.97
Shooting position	4.53-8.67	4.37-8.64
Use of rest	1.48-5.68	1.49-5.70
Comfort	1.19-1.19	1.18-1.18
Time available	2.19-2.69	2.25-2.75
Point of aim	1.20-1.38	1.20-1.38
Distance to target	1.71	1.71
Light	1.15-1.61	1.15-1.60
Weather	1.07-1.19	1.07-1.19

Wind strength	1.10-1.15	1.10-1.16
Wind angle	1.28-3.45	1.29-3.45
Known area	2.25-5.29	2.43-5.27
Habitat type	3.11-4.91	3.10-4.92
Ground vegetation	1.25-1.79	1.25-1.79
Concealment	5.86-6.46	6.27-6.85
Deer species	1.65-2.96	1.66-2.95
Deer sex	1.21-1.21	1.21-1.21
Deer age	1.18-1.37	1.18-1.38
Alone or group	1.53-1.62	1.53-1.62
Alert state	1.42-3.86	1.41-3.93
Deer orientation	3.07-5.47	3.06-5.43
