# Supplementary material for: Factors Associated with Shooting Accuracy and Wounding Rate of Four Managed Wild Deer Species in the UK, Based on Anonymous Field Records from Deer Stalkers
Source: PLoS One. 2014 Oct 15;9(10):e109698. doi: 10.1371/journal.pone.0109698 (PMC4198128; doi:10.1371/journal.pone.0109698)
Supplement: Form S1 — Instructions and form for collecting data on shots fired at deer. (PDF) [file pone.0109698.s005.pdf]

FORM 1 - KEY FOR COMPLETING RECORD OF SHOTS FIRED AT A DEER

General idea. Use one line for each shot - but use the same deer reference number for multiple shots at one animal. Insert a number(s) in each column that best describes the result or situation.

Deer number. Label your first deer '001' (you may need to record more than one shot at a deer), the second deer is '002' etc (Correlate with the "deer" form and keep a running record if you go onto another sheet.).

Shooting position. Lying / prone = **1**; Sitting / kneeling / crouching = **2**; Standing = **3**; High seat or vehicle = **4**.

Shooting rest. Flat rest (e.g. wall, bank, backpack**)** = **1**; Bipod = **2**; Stick(s) = **3**; High seat rail = **4**; Vertical rest (e.g. tree, post) = **5**; Off elbows (lying, sitting or kneeling) = **6**; Unsupported/ Freehand = **7**;

Stability. Comfortable/ steady = **A**; Uncomfortable/ unsteady = **B**.

Wind strength. 0 mph - 10 mp**h** = **1**; 11 mph - 20 mph = **2**; 21 mph - 30 mph = **3**; 31 mph - 40 mph = **4**.

Wind angle. Use the clock method to record where the wind is coming from, assuming that you are looking towards 12 o'clock. A wind blowing at right angle from left to right is from 9 o'clock / from behind you (unlikely in the field!) is from 6 o'clock. No wind = **0**.

Time available. Hurried = **1**; Very little time = **2**; Sufficient time = **3**; More than adequate time = **4**.

Point of aim. Chest (heart / lungs) = **1**; Low neck = **2**; High neck = **3**; Head = **4**.

Deer orientation. Use the clock method for the way the deer is facing - broadside with head facing right would be 3 o'clock; facing directly away from you is 12 o'clock.

Range: estimated. Estimate the range when you fired.

Range: measured. Pace or measure the exact distance afterwards.

FORM 1 - EXAMPLE

Notes. The example illustrates the following situation:

The stalker was lying down (1) and using a steady bipod (2,A) with a light breeze (1) blowing from left to right. He had plenty of time to take aim and shoot (4), aimed at the heart (1) with the deer facing side-on with its head to the right. He estimated the range as 100m and found it to be 90m when he paced to the spot after firing.

If you need to explain something just mark the entry with * and write an explanation on the back of form.

FORM 1 - DETAILS OF EACH SHOT FIRED AT A DEER

Stalker ID /Registration Number: ____________________________

**Conditions under which shot was taken**									
Deer Ref. No.	Shooting position	Shooting rest used	Wind strength	Wind angle	Time available	Point of aim	Deer orientation	Range	
								Estimate (m)	Measured (m)
00X	1	2,A	1	9 o'clock	4	1	3 o'clock	100m	90m
