# Supplementary material for: Factors Associated with Shooting Accuracy and Wounding Rate of Four Managed Wild Deer Species in the UK, Based on Anonymous Field Records from Deer Stalkers
Source: PLoS One. 2014 Oct 15;9(10):e109698. doi: 10.1371/journal.pone.0109698 (PMC4198128; doi:10.1371/journal.pone.0109698)
Supplement: Form S2 — Instructions and form for collecting data on deer fired at. (PDF) [file pone.0109698.s006.pdf]

FORM 2 - KEY FOR COMPLETING RECORD OF DEER FIRED AT WHILE STALKING.

General Idea. Use one line for each shot taken at a deer. Insert number/letter(s) in each column that best describes the result or situation – explain on reverse if complicated.

Deer Ref No. Correlate with the "Shots" form.

Species. Red = **1**; Fallow = **2**; Sika = **3**; Roe = **4**; Muntjac = **5**; CWD = **6**.

Sex. Stag / Buck = **1**; Hind / Doe = **2**.

Age. Under 1 year = **1**; Young e.g. 1- 2 years old = **2**; Mature Adult = **3**; Old = **4**.

Alone/ Group. Alone = **1**; In a small group (2-4) = **2** In a larger group (5-10) = **3**; In a herd (10+) = **4**.

Alert State. Unaware of you = **0**; Alert = **1;** Suspicious = **2**; Looking intently at you = **3**; About to run = **4;** On the move = **5**.

Light Conditions. Very bright = **0**; Quite bright = **1**; Dull = **2**; Quite Dark = **3**; Twilight = **4**; Night Shooting = **5**.

Weather. Fine = **0**; Raining = **1**; Hailing/Snowing = **2**; Fog /Mist = **3**.

Known Area. Very well known (stalked often) = **0**; Well known (stalked regularly) = **1**; Known (stalked occasionally) = **2** Not well known = **3**; Unknown/new area = **4**.

Habitat Type. Bare Hillside = **0**; Open/Broken Ground = **1**; Fields = **2**; Small woods/ fields = **3**; Open Woodland = **4**; Thick Woodland = **5**.

Ground Vegetation. Bare Ground = **0**; Vegetation < 0.5 m high = **1**; Vegetation < 1m high = **2**; Vegetation 1 - 2 m high; = **3**; Woodland > 1m high; = **4**; Thicket = **5**.

Concealment. Deer unobscured = **A**; Deer partially obscured = **B**; Deer heavily obscured = **C**.

Rifle Calibre. e.g. .222 / .243 / 7mm / .308 etc.

Bullet Weight. 50gr / 100gr / 140gr etc.

Killed / Hit / Lost / Missed. Record what happened to the deer: Killed with 1 shot = **1**; Hit but other shot(s) required to kill it [list results of each shot on Form 1] = **2**; Hit but not retrieved or fired at again (lost) = **3**; Clean miss = **4**.

"Dead" is defined as no blink reaction when an eyeball is touched. If you cannot reach the deer to test for this within 2 minutes, record the fact with perhaps a note on reverse as to why. e.g. I waited 5 minutes as deer not seen to drop in thick cover/ Deer was seen to drop but I took more than 2 minutes to reach it across a gorge. If you hit a deer and then finish it off quickly with a second shot - record it as **"hit"**.

FORM 2 – EXAMPLE OF DEER FIRED AT

Notes. The example illustrates the following situation:
A red deer (1) stag (1) yearling (2) from a small group (2) was suspicious but not looking at the stalker (2) on a dull day with light rain (2,1) in a fairly well known area (1) with broken ground (1) standing in short bracken (2) but the deer was clear (A). .308/150gr rifle/ammo was used, killed the deer with a single shot (1).

Correlation with the “shots” (Form 1) will probably explain the full picture and history of each event to researchers but if it is really complicated, please explain on reverse!

FORM 2 - DETAILS OF EACH DEER FIRED AT
(WHETHER KILLED OR NOT)

Stalker ID /Registration Number: ______________________

**Details of the Deer**						**Environmental Conditions**				**Outcome for Deer**	
Deer Ref. No.	Type of Deer	Sex	Age	Alone or Group	Alert State	Light/ Weather	Known area	Habitat Type	Ground Vegetation / Conceal-ment	Rifle/ Bullet Weight Used	Killed/ Hit/ Lost/ Missed
00X	1	1	2	2	2	2,1	1	1	2A	308/ 150 gr	1
